# The Role of Furin in the Pathogenesis of COVID-19-Associated Neurological Disorders

**DOI:** 10.3390/life14020279

**Published:** 2024-02-19

**Authors:** Gunel Ayyubova, Sergiy G. Gychka, Sofia I. Nikolaienko, Fada A. Alghenaim, Tadahisa Teramoto, Nataliia V. Shults, Yuichiro J. Suzuki

**Affiliations:** 1 Department of Cytology, Embryology and Histology, Azerbaijan Medical University, Baku AZ1022, Azerbaijan; gunel.ayubova@gmail.com; 2 Department of Pathological Anatomy, Bogomolets National Medical University, 01601 Kyiv, Ukraine; gychka59@gmail.com (S.G.G.); s.nik@nmu.ua (S.I.N.); 3 Department of Pharmacology and Physiology, Georgetown University Medical Center, Washington, DC 20007, USA; fa485@georgetown.edu; 4 Department of Microbiology and Immunology, Georgetown University Medical Center, Washington, DC 20007, USA; tt54@georgetown.edu; 5 Department of Biology, Georgetown University, Washington, DC 20007, USA; ns1015@georgetown.edu

**Keywords:** Alzheimer’s disease, angiotensin II, angiotensin-converting enzyme 2, COVID-19, brain, dementia, furin, neurological disorders, SARS-CoV-2, spike protein

## Abstract

Neurological disorders have been reported in a large number of coronavirus disease 2019 (COVID-19) patients, suggesting that this disease may have long-term adverse neurological consequences. COVID-19 occurs from infection by a positive-sense single-stranded RNA virus called severe acute respiratory syndrome coronavirus 2 (SARS-CoV-2). The membrane fusion protein of SARS-CoV-2, the spike protein, binds to its human host receptor, angiotensin-converting enzyme 2 (ACE2), to initiate membrane fusion between the virus and host cell. The spike protein of SARS-CoV-2 contains the furin protease recognition site and its cleavage enhances the infectivity of this virus. The binding of SARS-CoV-2 to the ACE2 receptor has been shown to downregulate ACE2, thereby increasing the levels of pathogenic angiotensin II (Ang II). The furin protease cleaves between the S1 subunit of the spike protein with the binding domain toward ACE2 and the S2 subunit with the transmembrane domain that anchors to the viral membrane, and this activity releases the S1 subunit into the blood circulation. The released S1 subunit of the spike protein also binds to and downregulates ACE2, in turn increasing the level of Ang II. Considering that a viral particle contains many spike protein molecules, furin-dependent cleavage would release many free S1 protein molecules, each of which can downregulate ACE2, while infection with a viral particle only affects one ACE2 molecule. Therefore, the furin-dependent release of S1 protein would dramatically amplify the ability to downregulate ACE2 and produce Ang II. We hypothesize that this amplification mechanism that the virus possesses, but not the infection per se, is the major driving force behind COVID-19-associated neurological disorders.

## 1. Introduction

Coronavirus disease 2019 (COVID-19) is caused by the severe acute respiratory syndrome coronavirus 2 (SARS-CoV-2), a positive-sense single-stranded RNA virus of the Coronaviridae family, and had a devastating impact worldwide. As of December 2023, there have been over 770 million recorded cases and almost 7 million deaths globally, although the actual numbers are believed to be higher. This pandemic has been one of the deadliest in history, making COVID-19 a leading cause of death [1]. While the pandemic has now officially been declared over, many still suffer from medical issues related to this virus, such as long COVID or post-acute sequelae of SARS-CoV-2 infection (PASC).

Emerging evidence suggests that although SARS-CoV-2 was initially thought to be primarily a respiratory illness, it has the ability to infiltrate the central nervous system, where it can cause a variety of impairments—from non-specific symptoms, such as confusion, anosmia, and anxiety [2,3,4], to serious long-term neurological complications, including cognitive impairments, cerebrovascular diseases, demyelinating pathologies, encephalopathy, stroke, etc. [5,6]. Problems affecting the central nervous system have been reported in more than ∼35.6% of total COVID-19 cases [7]. Additionally, hippocampal shrinkage, reduction in brain size, and neurodegeneration have been reported following SARS-CoV-2 infection [8,9]. Therefore, it is crucial to shed light on SARS-CoV-2 invasion and its impact on the central nervous system. SARS-CoV-2 has been shown to replicate in neuronal U251 cells, supporting the idea that the virus may play a part in the development of neurological lesions [10]. SARS-CoV-2 viral RNAs and proteins were found in anatomically diverse brain areas and cerebrospinal fluid, providing indications of SARS-CoV-2 neuroinvasion and neurotropism [11,12]. However, despite these findings, it is unknown whether the neurological impairments linked to SARS-CoV-2 are the result of direct viral and/or spike protein action or are instead a result of hypoxia, a surge of pro-inflammatory cytokines driven by infection, or vascular and blood–brain barrier abnormalities [13,14,15,16]. There are also reports showing that the SARS-CoV-2 proteome can be assembled into neurotoxic amyloids, causing neurological symptoms that appear after infection [17].

Evidence suggests that the malfunctioning of the renin–angiotensin system components in the brain contributes to the neurological symptoms of COVID-19 [18,19]. SARS-CoV-2 infects host cells through the interactions between the membrane fusion protein of the virus, the spike protein, and its human host receptor, angiotensin-converting enzyme 2 (ACE2) [20]. ACE2 physiologically functions to convert angiotensin II (Ang II) into angiotensin 1–7 (Ang 1–7) [21], thereby degrading Ang II. Ang II, a decapeptide that is produced from Ang I in the renin–angiotensin–aldosterone system, is the major vasoconstrictor and plays a crucial role in the development of many diseases, including neurological disorders.

The binding of the SARS-CoV-2 spike protein to ACE2 has been shown to activate multiple biologic mechanisms that result in the reduction of the expression of ACE2 in the plasma membrane, thereby decreasing the peptidase activity of ACE2 to convert Ang II to Ang 1–7, in turn increasing the levels of Ang II [22]. An increase in the level of Ang II activates the Ang II/Ang II receptor type 1 (AT1R) pathway, thereby accelerating pathogenic mechanisms including neurological complications and neurodegeneration [23,24]. It has been shown that the angiotensin-converting enzyme (ACE)/Ang II/AT1R axis is upregulated in neurodegenerative diseases, and causes oxidative stress, increased permeability of the blood–brain barrier, neuroinflammation, neurovascular dysfunction [25], and a reduction in cerebral blood flow, contributing to the development of Alzheimer’s disease [26]. Thus, medications that inhibit the renin–angiotensin system may reduce the risk of developing Alzheimer’s disease [26].

In the brain, the deleterious effects of the ACE/Ang II/AT1R pathway are counterbalanced by its alternative axes, which are ACE/Ang II/Ang II receptor type 2 (AT2R) and ACE2/Ang 1–7/Mas receptor, both of which have positive effects on cognition and memory. The counteracting and protective effects of the ACE2/Ang 1–7/AT2R and MAS receptor pathways are associated with the lowering of oxidative stress and inflammatory reactions, as well as vasodilation through the creation of nitric oxide and prostaglandins [27,28], in addition to antithrombotic effects and neuroprotection [29,30,31].

Neurological consequences seen in COVID-19 have also been linked to impaired neurotransmission in the central nervous system caused by SARS-CoV-2. Identifying the pathways of how SARS-CoV-2 affects the central nervous system should help develop effective treatment strategies and prevent its negative effects on neurocognition. We herein propose a novel mechanism in which spike protein-mediated effects may be amplified and contribute to COVID-19 associated neurological disorders.

## 2. The Hypothesis

Our hypothesis is that the furin protease-dependent cleavage of the SARS-CoV-2 spike protein and release of the circulating S1 subunit protein amplify ACE2 downregulation and subsequent Ang II production, thereby promoting neurological disorders seen in COVID-19 patients.

**Figure 1:**
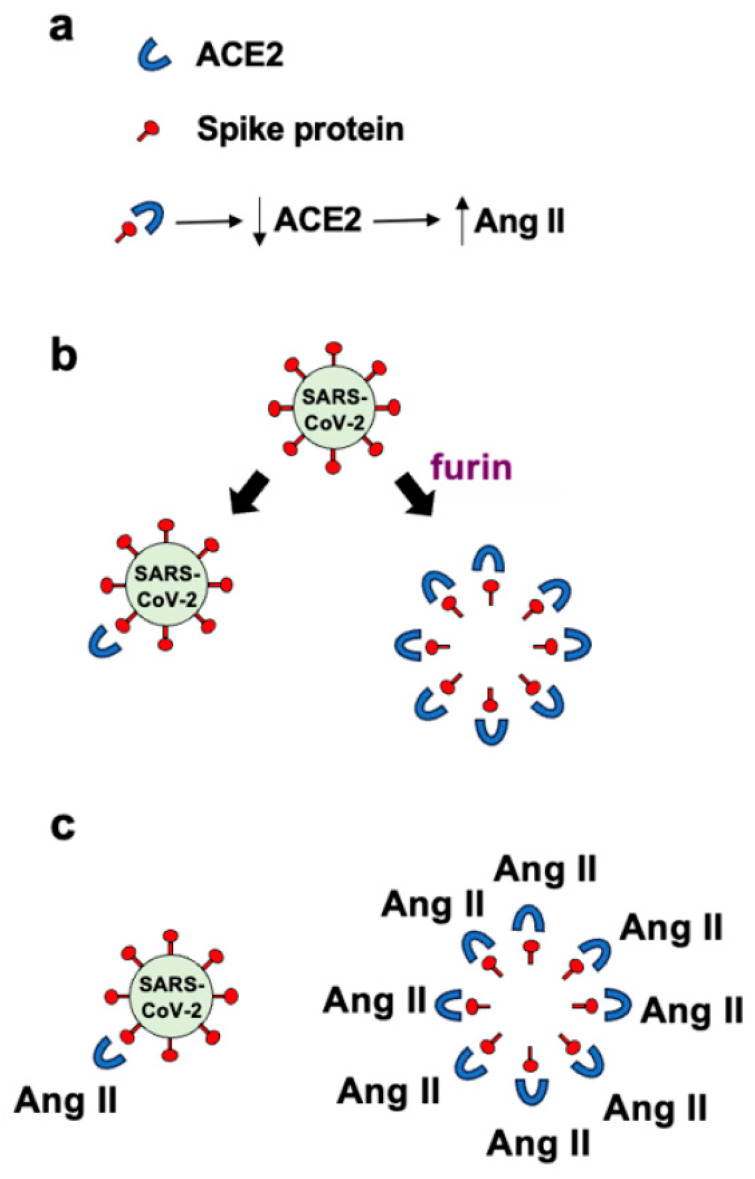
Schemes depicting the main hypothesis of this paper. (**a**) The binding of the spike protein to ACE2 downregulates (↓) the ACE2 protein, thus increases (↑) Ang II. (**b**) One spike protein molecule of one SARS-CoV-2 viral particle interacts with one ACE2 molecule, resulting in downregulating one ACE2 molecule per viral particle. If many spike protein molecules of a given viral particle are cut by the furin protease and come off the virus, multiple free spike protein molecules are produced, which can result in the binding of multiple ACE2 molecules per viral particle. This could result in many ACE2 molecules becoming downregulated. (**c**) The amplification of ACE2 downregulation dramatically increases the level of Ang II.

Panel (a) in Figure 1 depicts the binding of the spike protein to ACE2 to downregulate the ACE2 protein. The downregulation of ACE2 results in the reduced overall peptidase activity of this enzyme to degrade Ang II, thereby increasing the levels of Ang II, a major pathogenic mediator. Panel (b) of Figure 1 shows that it may be expected that the ratio of roughly one spike protein molecule of one SARS-CoV-2 viral particle interacts with one ACE2 molecule, resulting in downregulating one ACE2 molecule per viral particle. If many spike protein molecules of a given viral particle are cut by the furin protease and come off the virus, multiple (perhaps 50–100) free spike protein molecules are produced, which can result in the binding of multiple (50–100) ACE2 molecules per viral particle. This could result in 50–100 ACE2 molecules becoming downregulated. Panel (c) in Figure 1 shows that the amplification of ACE2 downregulation by furin-dependent cleavage of the spike protein forms free S1 protein that then dramatically increases the level of Ang II. Higher levels of Ang II are expected to produce more pathological conditions, including neurological damage. We propose that this furin-dependent amplification process contributes to the mechanism of COVID-19-associated neurological disorders.

## 3. Evaluation of the Hypothesis

This hypothesis follows up the previously described viral protein fragment theory of COVID-19 pathogenesis [32] which illustrated that, as humans are infected with SARS-CoV-2, the virus releases fragments of the spike protein that can target host cells without the rest of the viral component.

SARS-CoV-2 is a single-stranded RNA virus that attaches to the host cells through the interactions between the spike protein (the membrane fusion protein of this virus) and the host cell receptor ACE2, leading to the fusion of the viral and host cell membranes that allows the entry and subsequent replication of the virus. Although SARS-CoV-2 is a respiratory virus, other organs such as the brain are often affected, which raises questions about whether merely the infection and replication of the virus in the host cells alone are responsible for the pathologies associated with COVID-19.

**Figure 2:**
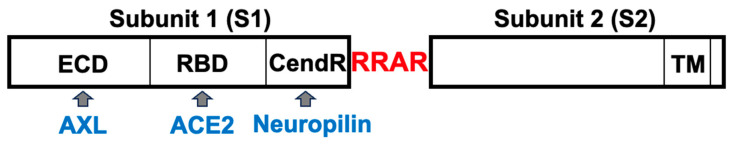
The structure of the SARS-CoV-2 spike protein. The spike protein consists of S1 and S2 subunits. The S1 subunit contains the RBD, which binds to ACE2, and the S2 subunit contains the transmembrane (TM) domain that anchors to the viral membrane. The S1 subunit also contains the extracellular domain (ECD) that binds to AXL and the CendR domain that binds to neuropilin 1. Between the S1 and S2 subunits is an RRAR amino acid sequence that is a consensus sequence where furin protease cleaves.

The SARS-CoV-2 spike protein is composed of two subunits: S1 and S2 (Figure 2). The S2 subunit contains the transmembrane domain (TM) and is anchored to the viral membrane. The S1 subunit of the spike protein sticks out of the viral particle and contains the receptor-binding domain (RBD) that interacts with the major host cell receptor of SARS-CoV-2, ACE2 [33,34]. During the virus entry to host cells, the spike protein is cleaved into S1 and S2 subunits mainly by transmembrane serine protease 2 (TMPRSS2) at the cell surface of lung epithelial cells. Proteolysis into S1 and S2 subunits by more ubiquitous enzymes such as the furin proprotein convertase also occurs and has been shown to enhance the infectivity of the SARS-CoV-2 virus [35].

In addition to ACE2 binding to the spike protein RBD of the intact virus to facilitate the viral entry, the S1 subunit of the spike protein can be cleaved off from the virus and released in the blood circulation by proteases such as furin. In fact, the circulating S1 protein has been detected in COVID-19 patients. Ogata et al. [36] used ultra-sensitive serial profiling Single-Molecule Array (Simoa) assays to quantitatively detect SARS-CoV-2 spike, the S1 subunit, and nucleocapsid antigens in the plasma of COVID-19 patients. The authors detected SARS-CoV-2 S1 and nucleocapsid antigens in 41 out of 64 COVID-19-positive patients. In a retrospective study of plasma samples collected from 63 patients in Boston, SARS-CoV-2 proteins including S1 spike protein were detected in the plasma of the majority of COVID-19 patients with long COVID conditions and were persistently detected at various time periods up to 12 months after diagnosis [37]. Further, widely used mRNA COVID-19 vaccines that encode for the full-length spike protein (S1 + S2) have also been shown to produce the circulating S1 protein [38,39,40]. Ogata et al. [38] again used the Single-Molecule Array (Simoa) assays to detect SARS-CoV-2 spike, the S1 subunit, and nucleocapsid proteins in the plasma of 13 mRNA–1273 vaccine recipients. Overall, 11 of 13 participants exhibited detectable levels of S1 subunit protein as early as one day after the first vaccine administration. The release of the S1 subunit of the spike protein from the COVID-19 vaccine could be due to furin-dependent cleavage of the S1 + S2 spike protein molecules that are expressed on the plasma membrane with the S1 side facing extracellularly after the administration of mRNA vaccines. Taking into consideration the experimental results showing that an intravenously injected S1 protein can easily cross the murine blood–brain barrier and enter the parenchymal tissue and interstitial fluid spaces of the brain [41], as well as its persistence in circulation long after infection, we can suggest that the circulating S1 is more likely to cause neurological complications than the virus itself.

The furin subtilisin-like eukaryotic endoprotease cleaves proteins at the consensus amino acid sequence Lys/Arg-X_n_-Lys/Arg [42]. It is a protein with a calculated molecular weight of 87 kDa and was named furin because it was in the upstream region of an oncogene FES, thus becoming known as the FUR (FES Upstream Region). Since it cleaves basic amino acid motifs, it is also known as the paired basic amino acid-cleaving enzyme (PACE). It is ubiquitously expressed with high levels found in the salivary glands, liver, and bone marrow. Physiologically, it functions to exert proteolytic activation of various hormones, growth factors, receptors, adhesion molecules, and enzymes. Its proteolytic substrates also include proteins of various bacterial toxins and viruses, including human immunodeficiency virus (HIV) and dengue virus. In mammalian cells, furin accumulates in the Golgi, and it can traffic to the plasma membrane, while C terminal proteolytic cleavage separates the transmembrane domain from the catalytically active domain that could occur in the extracellular space [42]. Using the fluorogenic substrate boc-Arg-Val-Arg-Arg-MCA, Vidricaire et al. [43] detected the endoproteolytic activity of secreted furin in the media of BSC40 cells overexpressing furin. As furin can occur in the extracellular space, the circulating S1 may be produced from SARS-CoV-2 as well as from COVID-19 vaccines by this enzyme.

The S1 subunit of the spike proteins of both SARS-CoV-2, which caused COVID-19, and SARS-CoV, which caused severe acute respiratory syndrome (SARS), contains the RBD that binds to ACE2. Since the 2002 SARS outbreak, research has shown that the spike protein binding to its host cell receptor ACE2 results in the downregulation of ACE2, in turn increasing the major pathogenic mediator Ang II. In mice, Kuba et al. [44] reported in 2005 that SARS-CoV infection, as well as injection with the recombinant SARS-CoV spike protein, reduces ACE2 expression. Importantly, the authors showed that the worsening of acute lung failure in this infection is primarily caused by SARS-CoV spike protein-mediated ACE2 downregulation. In these mice, the spike protein increased Ang II, and the angiotensin receptor inhibitor losartan attenuated the spike protein-induced enhancement of lung injury. In HEK293 cells, the SARS-CoV spike protein RBD was found to be internalized together with ACE2 [45].

After the COVID-19 pandemic started, Bayati et al. [46] found that SARS-CoV-2 undergoes clathrin-mediated endocytosis in HEK293T cells. The SARS-CoV-2 infection downregulates ACE2 in Syrian golden hamsters and in cultured HEK293A cells transfected with ACE2 by inducing clathrin-dependent endocytosis and degradation in the lysosome [47]. Expression of GFP-tagged ACE2 in HEK293T cells also demonstrated the internalization of ACE2 in response to the recombinant RBD protein treatment [48]. Using structured illumination microscopy, endocytosis of the SARS-CoV-2 spike protein RBD–ACE2 complex was visualized in living cells [49]. These results provide evidence that the binding of the spike protein to ACE2 results in endocytosis-mediated internalization of the spike protein–ACE2 complex into the cells and the ultimate degradation of ACE2. Lei et al. [50] reported that Syrian hamsters infected with spike protein-expressing pseudovirus had reduced ACE2 protein expression in the lungs. Their experiments suggested a mechanism by which the spike protein increases redox stress, leading to AMPK deactivation, MDM2 upregulation, and ACE2 destabilization. In addition to the ACE2 protein downregulation mechanisms via spike protein-mediated internalization and degradation, Sui et al. [51] reported that the SARS-CoV-2 spike protein reduces the mRNA expression of *ACE2* in primary cells of lung bronchoalveolar lavage from naïve rhesus macaques. An interesting study by Gao et al. [52] similarly suggested that the internalized SARS-CoV-2 spike protein activates intracellular signals to degrade *ACE2* mRNA.

**Figure 3:**
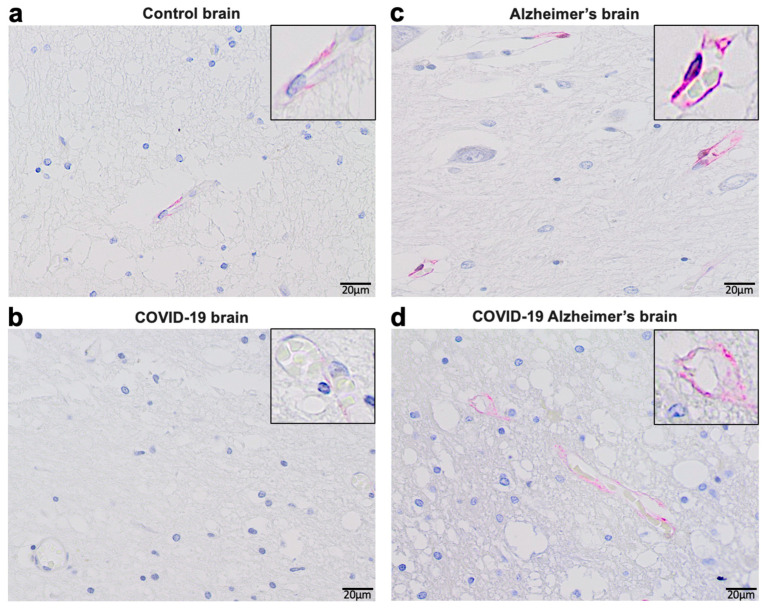
Immunohistochemical evaluations of ACE2 protein expression in human brains.

Consistent with these experimental findings, our immunohistochemical evaluations of human patients who died of COVID-19 showed reduced ACE2 protein expression by COVID-19 in patients both with and without Alzheimer’s disease (Figure 3). In the brains of patients without known neurological diseases, ACE2 protein expression was predominantly detected in capillary endothelium as shown in the pink stain (Panel (a)). Patients who died of COVID-19 showed downregulated ACE2 protein expression at a very low level (Panel (b)). Consistent with previously reported Western blotting and RT–PCR results obtained by our laboratory as well as others [53,54,55,56], ACE2 expression levels were higher in the brains of Alzheimer’s disease patients compared to controls as monitored by immunohistochemistry (Panel (c)). Even in Alzheimer’s brains, COVID-19 decreased the protein expression of ACE2, but only to a level that was higher than in the brains of COVID-19 patients without Alzheimer’s disease (Panel (d)). Thus, it can be speculated that COVID-19 decreases ACE2 expression in the brain via the actions of the spike protein, highlighting the importance of our hypothesis that furin would amplify the actions of the spike protein to downregulate ACE2.

### 3.1. Materials and Methods (Figure 3)

De-identified postmortem formalin-fixed paraffin-embedded human brain tissues obtained in Kyiv, Ukraine were cut into 5 μm thick sections. Slides were subjected to immunohistochemistry using an anti-ACE2 antibody (Rabbit Angiotensin Converting Enzyme 2 Monoclonal Antibody) purchased from MyBioSource (San Diego, CA, USA) and the Master Polymer Plus Detection System (Phosphatase and AP Chromogen) purchased from Vitro Master Diagnostica, Spain. Specimens were examined using a Leica BX 51 microscope, a Leica MC 190 digital camera, and the Leica LAS software (Leica Application Suite X 3.0.12) at a magnification of 400×.

### 3.2. Results (Figure 3)

Immunohistochemistry analysis using the ACE2 antibody showed that control brains from individuals without neurological diseases exhibited ACE2 protein expression in the capillary endothelium as shown in pink staining (Figure 3a). Patients who died of COVID-19 showed dramatically downregulated ACE2 (Figure 3b). Alzheimer’s disease patients showed upregulated ACE2 protein expression in the brain (Figure 3c). COVID-19 also downregulated ACE2 protein expression in the brains of patients with Alzheimer’s disease (Figure 3d).

## 4. Consequences of the Hypothesis

Since the physiological function of ACE2 is to degrade Ang II [57], the loss of ACE2 results in increased Ang II and associated pathologies. ACE2 is a monocarboxypeptidase that is mainly expressed in vascular endothelial cells, although its expression in human neurons has also been reported. Xu and Lazartigues [58] showed the expression of ACE2 in human pluripotent stem cell-derived neurons by immunohistochemistry. ACE2 substrates have hydrophobic or basic residues at the C-terminal end, preceded by a Pro–X–Pro sequence, albeit having one proline residue is sufficient for ACE2 activity. Ang II is an octapeptide with the sequence Asp–Arg–Val–Tyr–Ile–His–Pro–Phe and a hydrophobic phenylalanine at the C-terminus, preceded by a proline residue. ACE2 cuts the C-terminal phenylalanine residue from the Asp–Arg–Val–Tyr–Ile–His–Pro that is Ang 1–7.

It has been shown that the Ang II levels in the plasma samples from SARS-CoV-2-infected patients in Shenzhen, China was markedly elevated [59]. Also, a study of 82 non-hypertensive patients in Wuhan, China by Wu et al. [60] showed that plasma Ang II level was higher in COVID-19 patients than non-COVID controls. A study of 30 patients hospitalized due to COVID-19 conducted at the Clinics Hospital at the University of Campinas in Brazil by Camargo et al. [61] showed that patients with critical COVID-19 had higher Ang II levels than patients presenting with severe COVID-19. In this study, levels of ACE, ACE2, Ang 1–7, and Ang 1–9 were found to be similar in the two groups. A study at Istanbul University—Cerrahpasa Hospital in Turkey by Ipekci et al. [62] showed that serum samples from COVID-19 patients had significantly lower ACE2 levels than controls and increased Ang II levels.

If the action of the spike protein to downregulate ACE2 only occurs via an intact virus, one may envision that one viral particle may downregulate one ACE2 protein molecule. It is thought that a coronavirus particle may contain about 50–100 trimers of spike proteins [63] based on an electron cryomicroscopy study performed on SARS-CoV by Neuman et al. [64], depending on whether the spike protein or ribonucleoprotein spacing is used to calculate the surface area of a spike unit cell. Thus, 50–100 spike protein molecules could be produced from one viral particle by furin proteolytic activity, amplifying the ability to downregulate ACE2 and produce Ang II 50–100-fold.

This hypothesis is important because it suggests that this amplification mechanism conferred by furin contributes to the pathogenesis of neurological and other complications seen in COVID-19. As this theory becomes proven, testing of furin inhibitors may benefit patients suffering from neurological and other disorders due to SARS-CoV-2 infection as well as COVID-19 vaccines.

**Figure 4:**
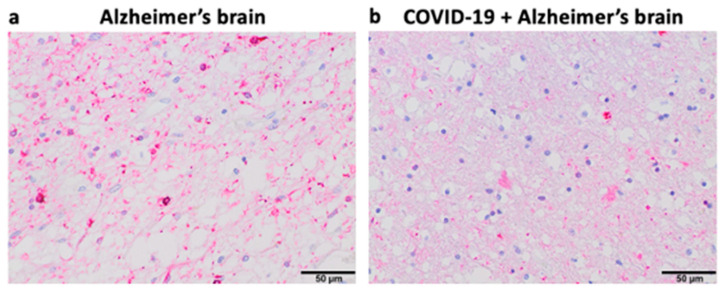
Immunohistochemistry of the brain of a patient who died of COVID-19 showing early signs of Alzheimer’s disease.

Our pilot study in Kyiv, Ukraine suggested that 6 out of 40 (15%) patients over 75 years of age who died of COVID-19 had early signs of Alzheimer’s disease. Figure 4 shows representative histology images of a patient who died of Alzheimer’s disease with strong Tau expression and pronounced brain atrophy (Panel (a)), as well as a patient who died of COVID-19 with milder expression of Tau and less pronounced brain atrophy, perhaps indicating an early stage of the development of Alzheimer’s disease (Panel (b)). In the context of this hypothesis paper, these findings raise the question of whether the furin-mediated amplification of the spike protein/ACE2 binding results in enhanced downregulation of ACE2 expression and subsequent elevation of Ang II and whether these events may lead to the appearance of early pathogenic events for Alzheimer’s disease in some COVID-19 patients.

### 4.1. Materials and Methods (Figure 4)

De-identified postmortem formalin-fixed paraffin-embedded human brain tissues obtained in Kyiv, Ukraine were cut into 5 μm thick sections. Slides were subjected to immunohistochemistry using the Tinto Tau antibody (purchased from Bio SB) and the Master Polymer Plus Detection System. Specimens were examined using a Leica BX 51 microscope, a Leica MC 190 digital camera, and the Leica LAS software at a magnification of 400×.

### 4.2. Results (Figure 4)

Panel (a) of Figure 4 shows an immunohistochemistry image of a patient who died of Alzheimer’s disease with pronounced expression of Tau (forming Tau tangles or deposits) and significant brain atrophy. Panel (b) of Figure 4 shows the immunohistochemistry image of a patient who died of COVID-19 with early-stage Alzheimer’s disease, weaker expression of Tau (less amounts of deposits), and less brain atrophy.

## 5. Alternative to the Stated Hypothesis

**Figure 5:**
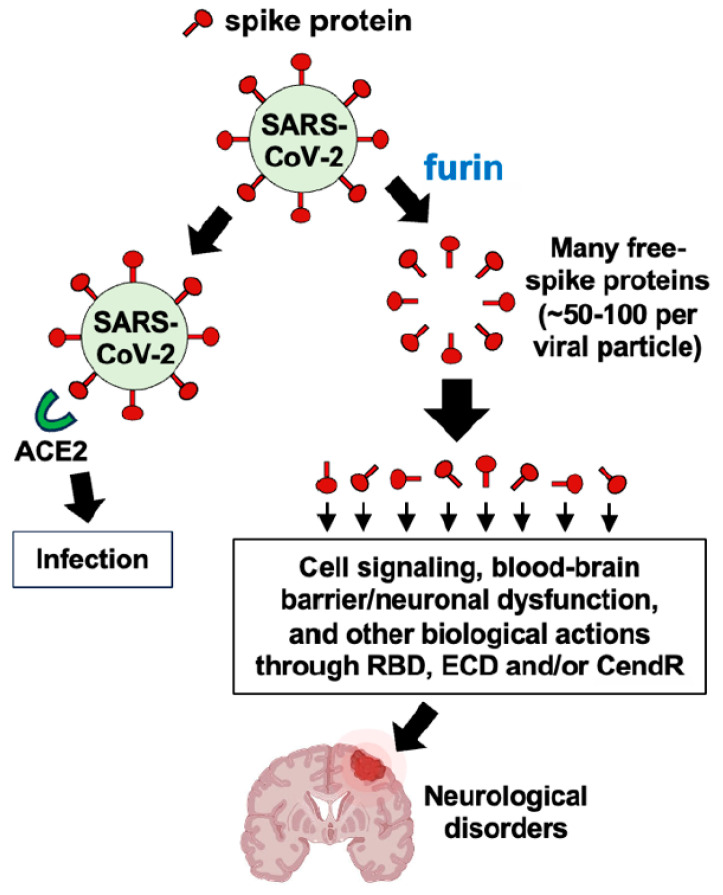
Schemes depicting the alternative hypothesis.

In Section 2 “The Hypothesis”, we provided a specific hypothesis based on ample reports in the literature describing that one major action of the spike protein is to downregulate ACE2. Thus, so far, we have focused our discussion on the actions of Ang II that would be expected to be increased as a consequence of ACE2 downregulation. However, the major thesis of this hypothesis paper is that the cleavage of spike protein molecules from the viral particle would amplify the actions of the spike protein by possibly 50–100 times, as described above. In addition to downregulating ACE2, the spike protein could elicit other biological actions, which would also be amplified through furin-dependent production of the S1 protein (Figure 5).

We describe above the possibility that the levels of Ang II are increased because of spike protein-mediated ACE2 downregulation based on several published reports supporting the role of Ang II in the pathogenesis of neurological diseases. However, there are reports refuting the role of Ang II in neurological disorders. The RADAR Trial, a double-blind, randomized, placebo-controlled, phase 2 trial concluded that 12 months’ treatment with losartan (an AT1R Ang II receptor blocker) was not effective in reducing the rate of brain atrophy in Alzheimer’s patients [65]. In mouse models of Alzheimer’s disease, the reduction in ACE2 levels and activity and increased Ang II did not exacerbate Alzheimer’s disease pathology [66]. Furthermore, while it is widely thought that AT1R mediates the pathogenesis of Alzheimer’s disease [67], Ang II can activate AT1R and AT2R, which could exert opposite biological effects.

Contrary to the widely reported concept that the spike protein downregulates ACE2, Aboudounya and Heads [68] proposed a signaling pathway in which the SARS-CoV-2 spike protein increases ACE2 expression, based on findings that the spike protein activates Toll-like receptor 4 [69,70]. We have also described the spike protein-activated cell signaling processes that may elicit biological responses independent of Ang II [71]. Further, the SARS-CoV-2 spike protein can cause blood–brain barrier and neuronal dysfunction either directly or via the activation of mast cells and microglia in the brain [72]. It should also be noted that the S1 subunit of the SARS-CoV-2 spike protein possesses, in addition to the RBD, the N-terminal extracellular domain (ECD) and the CendR domain. AXL receptor tyrosine kinase has been reported to bind to ECD [73] and neuropilin 1 binds to CendR [74,75]. Thus, the furin-dependent production of a number of free S1 protein molecules could amplify the actions of these factors as well.

## 6. Conclusions

This hypothesis paper presents a novel concept that the cleavage of the spike protein S1 subunit from the intact SARS-CoV-2 virus by proteases such as furin can result in a higher number of S1 protein molecules that can target ACE2 and/or other receptors in various tissues. The efficiency of such free S1 proteins in targeting ACE2 and other spike protein-binding proteins is expected to be 50–100 times higher than the intact spike proteins bound to the SARS-CoV-2 viral particles. Since spike protein/ACE2 interactions are known to downregulate the ACE2 protein through multiple mechanisms [22,65] and we observed that the brains of COVID-19 patients show reduced ACE2 expression (Figure 3), we hypothesize that this amplification mechanism would be an efficient way for the virus to promote and worsen pathogenesis in various organs, including the brain. This mechanism may affect patients who contracted COVID-19 and with post-acute sequelae of SARS-CoV-2 infection, as well as patients suffering from adverse neurological effects potentially caused by COVID-19 vaccines. If this hypothetic mechanism is proven to be correct, then the use of furin inhibitors could be one avenue for reducing pathologies associated with COVID-19.

## Data Availability

The data presented in this study are available on request from the corresponding author.
